# Altered Nutrient Composition of Lactose-Reduced Infant Formula

**DOI:** 10.3390/nu16020276

**Published:** 2024-01-17

**Authors:** Pari Mokhtari, Kelsey A. Schmidt, Mahsa Babaei, Michael I. Goran

**Affiliations:** Department of Pediatrics, The Saban Research Institute, Children’s Hospital Los Angeles, Los Angeles, CA 90027, USA; pmokhtari@chla.usc.edu (P.M.); kelschmidt@chla.usc.edu (K.A.S.); mbabaei@chla.usc.edu (M.B.)

**Keywords:** infant formula, lactose, formula feeding, macronutrients, micronutrients

## Abstract

This research comprehensively examines 88 infant formulas available in the US market, with an emphasis on their diverging nutritional attributes based on lactose content. We stratified formulas into three categories: lactose-free, lactose-reduced, and entirely lactose-based. The formulas’ nutritional content for 58 nutrients was obtained from the Nutrition Data System for Research (NDSR). Nutritional analysis revealed significant differences in nutrient composition across formula categories. For example, the results showed significant associations between the lactose content and glycemic index (GI) of the formula as well as 25 other nutrients. Specifically, we showed that for every gram of lactose per 100 g of formula that is removed, there was a 10.1% increase in GI (β = −10.12, *p* ≤ 0.000), a 19%,5%, and a 2% increase in added sugar (β = −0.19, *p* < 0.01), protein (β = −0.05, *p* < 0.001), and polyunsaturated fatty acids (β = −0.01, *p* < 0.01). The substitution of lactose in infant formulas significantly alters their nutritional profile, inducing changes in GI, added sugar, protein, and polyunsaturated fatty acids. These modifications have potential consequences for infant growth and metabolic responses and could influence long-term health trajectories. The clinical relevance of the composition differences between formulas should be further explored.

## 1. Introduction

Breastmilk is universally recommended for the first 6 months of life to optimize infant health and development [1]. Despite the proven benefits of exclusive breastfeeding, up to 74% of infants in the United States (US) receive some amount of formula before 6 months of age [2]. Infant formula feeding is an important early life nutritional exposure and these formulas are mostly characterized by macronutrient composition, including carbohydrates, protein sources, protein hydrolyzation, and fat sources [3]. The primary carbohydrate in human milk and milk-based infant formulas is lactose [4]. However, in recent years, non-lactose carbohydrates have been increasingly used in infant formulas [5], primarily due to an increased concern about the possibility of lactose intolerance in infants [5]. While lactose reduction or elimination is warranted in certain medical conditions such as prematurity [6], short bowel syndrome, and galactosemia [7,8], the majority of healthy infants have the ability to digest lactose in human milk [3]. Choosing a lactose-free or lactose-reduced infant formula, when not medically indicated, unnecessarily alters the infant nutrient profile. Specifically, the most common carbohydrates replacing lactose in these formulas are glucose-derived polymers such as corn syrup, brown rice syrup, and maltodextrin or sucrose, which have a higher glycemic impact and are metabolized differently than lactose [3,5]. This can have lasting impacts as carbohydrates are the primary source of energy for infant growth and development, and the quantity and quality of carbohydrates consumed early in life can influence the child’s metabolic profile [9]. Further, early nutrition has also been shown to have a long-lasting influence on an infant’s immune, brain, and behavioral function and development [5].

Infants in the United States are being exposed to non-lactose carbohydrates and hypoallergenic formulas at levels that are considered to be excessive and unnecessary [3]. A recent study examined the source of macronutrients found in infant formula purchased from 2017 through 2019 [3]. Out of all the formulas purchased in the US, the average carbohydrate composition was approximately 52.7% lactose, 42.3% glucose, and 5.0% sucrose. Additionally, 59.0% of the purchased formula was lactose-reduced, and 28.5% was lactose-free [3]. This emphasizes the importance of evaluating the carbohydrate content and composition in infant formulas, ensuring that they adequately align with the nutritional needs of infants.

The nutrient composition of infant formulas, while regulated, can vary greatly by formula brand and type. Given the prevalence of formula use, the diverse range of formulas available on the market, and the known health impacts of early nutrient intake, it has become increasingly important to examine the nutrient profile of available infant formulas. However, little is known about the nutrient composition of infant formulas currently offered in the US market, and whether the nutrient composition differs by formula type, such as lactose-free or lactose-reduced formulas. Therefore, this study aimed to compare whether the lipid profile, protein, and carbohydrate content of infant formulas differ by their lactose content. 

## 2. Materials and Methods

This observational study was conducted from May to June 2023 in Los Angeles, California. The nutrient composition of infant formulas including ready-to-feed, prepared from powder, prepared from concentrate, and pediatric drinks was considered in this analysis. Dietary intake data were collected and analyzed using the Nutrition Data System for Research (NDSR) 2021 software version, developed by the Nutrition Coordinating Center (NCC), University of Minnesota, Minneapolis, MN, USA [10]. A total of 88 infant formulas marketed in the US were utilized for this research based on nutrient information available in the NDSR. The NDSR obtains its information from various sources, including label claims and manufacturer data. To address missing nutrient values, NCC database scientists use careful procedures to estimate values from similar foods, different forms of the same food, associated nutrients, and manufacturer-provided data. This ensures a comprehensive and accurate nutrient database for research purposes. The nutrient composition of infant formulas was calculated using the following procedure: the NCC Food calculation program determined the amount of each ingredient in each formula through a linear optimization algorithm, which estimates the number of ingredients in each formula that most closely approximates the nutrient profile reported on the Nutrition Facts panel. The manufacturer scoop amount, preparation instructions, and gram weight per 100 calories of different forms of the infant formula were used to standardize nutrient measurements and to calculate the nutrients. Both the macro- and micronutrient contents of each type of formula—energy, protein, lipid, carbohydrate, vitamins, and minerals—were expressed as the amount per 100 kcal of formula milk for each ingredient. We compared a total of 58 nutrients as well as the energy and glycemic index (GI) per 100 g of each formula. The formula categorization and nutrient comparison flow chart is included in Supplemental Figure S1.

### 2.1. Formula Subgroup Categorization

The carbohydrate source for each formula was classified as lactose, sucrose, or glucose-derived (including corn syrup, corn syrup solids, brown rice syrup, glucose syrup solids, and maltodextrin). Infant formula was then grouped into lactose-free (carbohydrate source < 1% lactose), reduced lactose (carbohydrate source < 100% lactose), or 100% lactose formula.

### 2.2. Nutrient Information

This section provides definitions for the nutrient components and GI definition found in the NCC Food and Nutrient Database.

### 2.3. Sugars

Sugars contain individual monosaccharides including glucose, fructose, and galactose, and disaccharides such as sucrose, lactose, and maltose. Added sugars are the sugars and syrups that are added to formulas during processing and preparation. Added sugars exclude naturally occurring mono- and disaccharides such as the lactose in milk or fructose in fruit. White sugar (sucrose), brown sugar, powdered sugar, honey, molasses, pancake syrup, corn syrups, high fructose corn syrups, inverted sugar, inverted syrup, malt extract, malt syrup, fructose, glucose (dextrose), galactose, and lactose are the ingredients labeled as “added sugar” in the NCC database, and are in alignment with the 2020 Dietary Guidelines for Americans [11].

### 2.4. Glycemic Index

The glycemic index (GI) is a ranking of carbohydrate foods based on their effect on postprandial glycemia compared with the effect produced by a reference food, either glucose or white bread [12]. GI is determined by an in vivo test in which subjects consumed a test food containing 50 g of available carbohydrates. Blood glucose levels after 2 h are expressed as a percent of the glucose response in the same subject after consumption of 50 g of available carbohydrate from the reference food. Foods with a GI (glucose reference) of 55 or less are classified as low glycemic foods, those with a GI of 70 or above are high glycemic foods, and those between 55 and 70 are medium glycemic foods [13]. The GI value reported in the NDSR for each formula was calculated from the GI of each carbohydrate ingredient and weighted by the available carbohydrates of each ingredient in that formula. The steps that were used to calculate the GI for the formula from its ingredients are described in the NDSR manual [10].

### 2.5. Fat Components

Fatty acids are grouped as saturated (no double bonds), monounsaturated (one double bond), and polyunsaturated (two or more double bonds). Values do not include the glycerol portion of the triglyceride or other fat-related compounds. Therefore, the sum of fatty acids is less than the total fat in the food.

### 2.6. Protein Components

The protein component of infant formula includes animal protein from animal products; vegetable protein is the amount of protein contributed by soy products.

### 2.7. Statistical Analysis

Descriptive analyses were performed to assess variable distributions. Stacked bar plots were used to display the average percent nutrient composition across three lactose categories for each type of infant formula. These categories encompass lactose-reduced, lactose-free, and 100% lactose formulas. Linear regression analyses were used to examine how the nutrient composition was associated with the lactose content of infant formula in different subgroups. Descriptive statistics are presented as mean ± standard deviation (SD). All statistical analyses were carried out in R (Version 4.1.1). The statistical significance of regression models was determined at *p* < 0.05.

## 3. Results

### 3.1. Mean Difference Analysis among Different Formula Groups

We compared the nutritional content of infant formulas categorized into three different groups: lactose-free, lactose-reduced, and 100% lactose. The nutritional profiles significantly differed based on the three lactose-derived formula types (Table 1). The comparison of mean values among various formula types reveals a consistent trend. Among the 28 significantly different nutrients and glycemic measures, most demonstrated elevated levels in the lactose-free group. The exception was observed in saturated fatty acids, which exhibited the highest concentration in the 100% lactose group. For example, the results showed that the lactose-free formula had significantly higher gram added sugar per 100-g formula (mean = 5.29, SD = 1.87, *p* ≤ 0.01) compared to both lactose-reduced formula (mean = 4.37, SD = 1.63, *p* ≤ 0.01) and 100% lactose formula (mean = 2.41, SD = 2.41, *p* ≤ 0.01).

**Figure 1:**
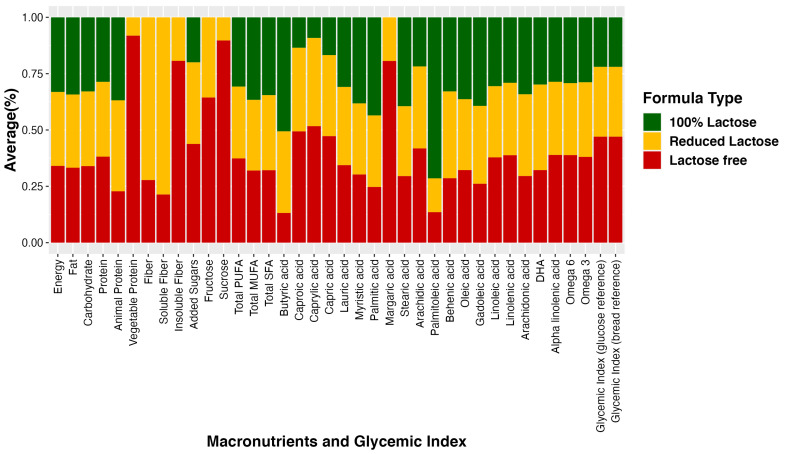

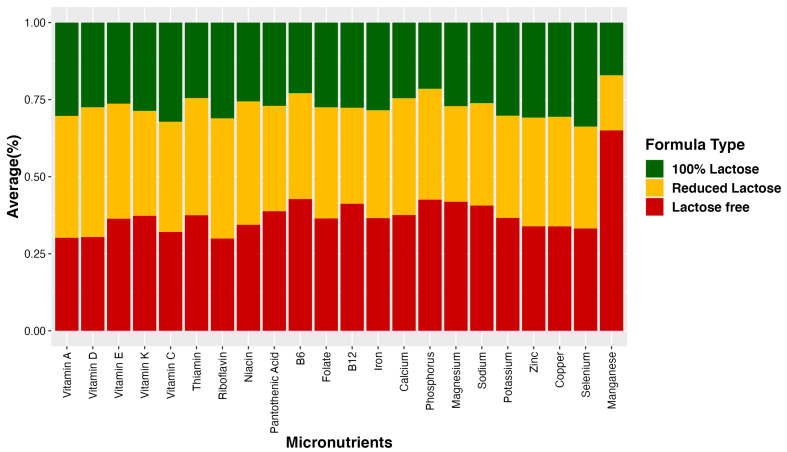
Average percent nutrient composition in three groups of infant formula. The bar graph illustrates the average percentage of nutrient composition in three distinct groups of infant formula: lactose-free, lactose-reduced, and 100% lactose formulas. Each bar represents the proportion of specific nutrients in each formula type, providing a visual comparison of their relative composition.

**Figure 2:**
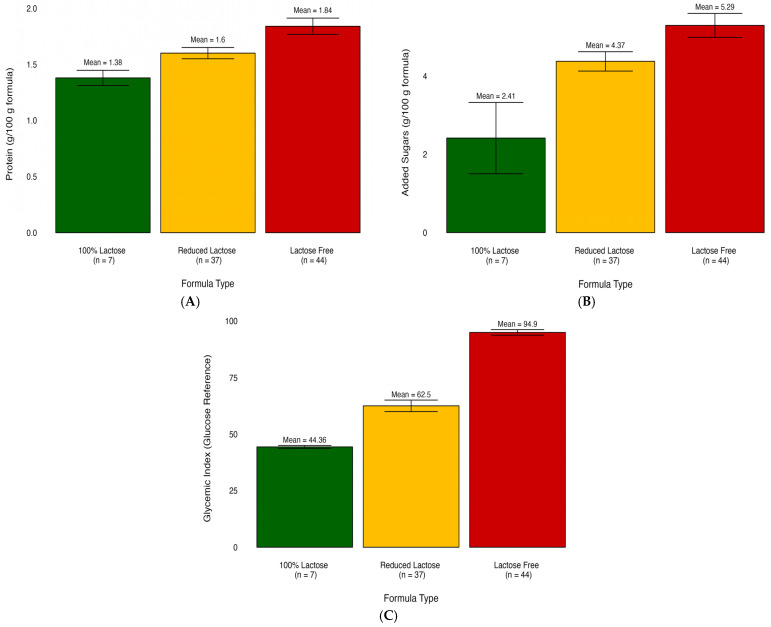
Bar plots illustrate the mean differences of nutrients in infant formula subgroups. (**A**) Comparison of added sugar content across formula types. (**B**) Comparison of protein content across formula types. (**C**) Comparison of glycemic index across formula types.

For visualization purposes, the average nutrient composition differences are highlighted in Figure 1, which uses a percent stacked bar chart to display the evolution of the proportion of each nutrient across lactose-free, lactose-reduced, and 100% lactose formulas. We further used bar graphs to show how certain nutrients vary on average among different infant formula groups (Figure 2). These graphs illustrate differences in added sugar content, protein content, and glycemic index across the various formula types.

### 3.2. Linear Regression Analysis: Nutrient–Lactose Concentration Associations

**Figure 3:**
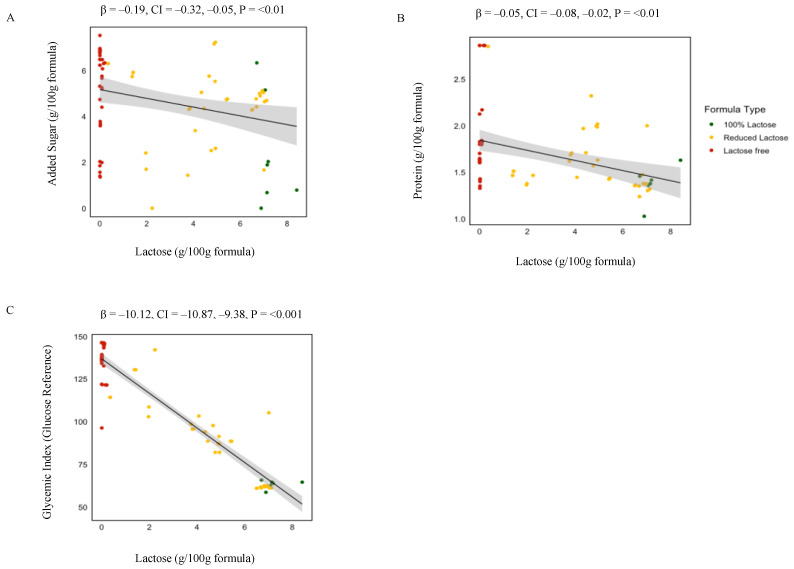
Linear regression analysis of lactose concentration and nutrient composition in infant formulas. This figure presents the associations between lactose concentration and the levels of added sugar (**A**), protein (**B**), and glycemic index (**C**) in 100% lactose (red), reduced lactose (blue), and lactose-free (green) formulas. Beta values and *p*-values are derived from linear regression analyses examining changes in added sugar, protein, or the glycemic index per 1 g increase in lactose concentration across formulas.

There were significant associations between GI as well as 25 out of the 58 nutrients and lactose content (Table 2). These results further confirmed the associations of gram nutrient intake with differences in lactose concentration of the formulas. Specifically, as shown in Figure 3 gram added sugar (*p* ≤ 0.01), gram protein (*p* ≤ 0.01), and the average GI (*p* ≤ 0.001) increased by 19%, 5%, and 10.1% for 1 g decrease of lactose/100 g of formula. In addition, the grams of total polyunsaturated fatty acids (β = −0.01, *p* ≤ 0.01) as well as essential fatty acids including Omega-3 (β = −0.001, *p* = 0.01) and Omega-6 (β = −0.01, *p* ≤ 0.01) increase for 1 g decrease of lactose/100 g of formula.

## 4. Discussion

The present study aimed to examine whether the nutritional profile of infant formulas differed based on their lactose content. Our findings revealed significant differences in the nutritional composition among the three lactose-derived formula types, namely lactose-free, lactose-reduced, and 100% lactose formulas. Formula with reduced lactose content was associated with higher levels of added sugar, protein, GI, and specific types of fatty acids. While higher levels of specific fatty acids like Omega-3 and Omega-6 may have beneficial effects on brain development and immune function in infants, the presence of increased added sugar and protein, and higher GI raises concerns about possible adverse effects on metabolic health and long-term health outcomes.

Lactose-reduced or lactose-free infant formulas commonly replace lactose with alternative carbohydrate sources, such as corn syrup solids (CSSFs), which are metabolized differently and have a higher GI [14,15]. In the US, infant formula composition is regulated by the FDA, which does not place limits on the usage of approved non-lactose carbohydrates. However, the Dietary Guidelines for Americans (2020–2025) [12] and the American Heart Association [16] recommend no added sugars in the first two years of life, emphasizing the importance of careful consideration of formula composition and the potential impact of added sugars on infant health.

Our results indicate that the selection of a 100% lactose versus a lactose-reduced or lactose-free formula has important implications for infant health given the differences observed in added sugar content and GI. One significant finding is the inverse relationship between lactose concentration and added sugar content. Added sugar levels increase by 19% for every 1 g reduction in lactose per 100 g of formula which raises concerns, given the growing evidence about childhood obesity and related health risks [17]. Excessive sugar intake during infancy has been linked to an increased risk of obesity and metabolic disorders later in life [18].

Recent research shows that glucose-based CSSF formulas caused significant alterations in postprandial metabolism compared to lactose-based formulas [19]. Furthermore, lactose is metabolized differently than sucrose, and the presence of sucrose in formulas has been associated with potential negative outcomes [3]. Studies have found that exclusively formula-fed infants or infants fed formula for longer durations had an increased risk of obesity compared to breastfed infants [20,21]. Further, the intake of formulas with high glycemic carbohydrate sources elevates the risk of obesity, attributing to adverse metabolic programming [21], increasing preference for sweet tastes [22], rapid weight gain [23], and alteration in gut microbiome composition [24,25].

One animal study shows that increased consumption of carbohydrates in the immediate postnatal time results in hyperphagia, hyperinsulinemia, and weight gain in rats [26]. In addition, higher intakes of carbohydrates with a low GI have been shown to improve insulin and glucose regulation in infants [9]. Furthermore, glucose-containing formulas cause different postprandial metabolic responses than lactose-containing formulas, notably a change in blood glucose levels in infants [19]. By opting for formulas with higher lactose content, caregivers can potentially reduce infants’ exposure to added sugars and support healthier growth trajectories. Despite these potential risks, no data exist regarding the long-term effects of sucrose or glucose polymer consumption in infancy from infant formula.

The lipid profile of human milk is complex, containing more than 200 fatty acids, with a lower concentration of saturated fatty acids, a higher content of oleic and linoleic acids, and essential fatty acids which are required for various physiological functions essential in promoting proper infant development [27]. Infant formula is a widely used human milk substitute designed to meet the nutritional needs of infants. While infant formula’s nutrient requirements are regulated, there is room for nutrient compositional differences among formula brands and types. This includes differences in lipid content. Therefore, the lipid profile of infant formulas is a critical determinant of infant health and development and long-term health outcomes [27]. For example, the concentration of polyunsaturated fatty acids (PUFA), such as Omega-3 and Omega-6 fatty acids, impacts optimal brain development, visual acuity, and immune function in infants [28]. In this study, we found that nutritional lipid composition differed by infant formula type. Specifically, our findings revealed that PUFA levels increase by 2% for every 1 g reduction in lactose per 100 g of formula. These variations in the PUFA levels among the different formulas could have major implications for neurodevelopment and long-term cognitive and visual outcomes [28]. This is especially significant as PUFAs play essential roles in cellular membranes, particularly within the brain, and act as precursors for various metabolites with distinct impacts on inflammation and the growth of neurons [28].

The impact of lactose concentration on protein content is another noteworthy result. The use of high-protein formula during the first two years of life, beyond meeting infants’ needs, has been associated with an increased risk of obesity and overweight in later childhood [29]. In addition, excessive protein intake can impact kidney function, as evidenced by research indicating that infants fed high-protein formulas exhibited elevated kidney volume and serum urea levels at 6 months compared to those on lower-protein formulas [30]. The ongoing debate on optimal infant formula protein content is shaped by the current European standards (1.8–2.5 g protein/100 kcal), with scientific societies setting a minimum (1.8 g/100 kcal) [30,31]. In this study, lactose-free formulas were found to have a protein level of 1.84 g per 100 g or approximately 2.6 g per 100 kcal of infant formula. This protein amount slightly exceeds the daily protein requirement for infants when considering their body weight, frequency of feeding, and daily formula intake [32]. This emphasizes the delicate balance required in formula design to ensure optimal protein intake for infant growth and development, while also considering the potential risks of excessive protein consumption in early life, which has been linked to an increased susceptibility to childhood obesity [33]. Most infant formulas are formulated with cow’s milk proteins and have fixed protein levels, which can be higher than those found in breast milk [34]. In contrast, the protein content in breast milk varies week by week to meet the changing needs of infants, particularly in the early stages of life. For example, breast milk at three months contains approximately half the protein content (0.8–1.0 g/100 mL) compared to the first weeks of life (1.4–1.6 g/100 mL) [34].

The early development of the infant gut microbiome is a complex and dynamic process that can be influenced by various factors, including dietary components [35]. One area of interest is the potential impact of sugar consumption and protein content in infant formula on the composition and diversity of the infant gut microbiome. It has been hypothesized that high sugar consumption during infancy, such as through the consumption of infant formulas with added sugars, may disrupt the balance of the gut microbiome [36]. While research in this area is still emerging, it is plausible that high sugar intake in early life may promote the growth of certain bacteria that thrive on sugar, potentially leading to an imbalance in the gut microbiota [37]. This disruption may have implications for infant health, including metabolic health and the risk of developing obesity later in life [37].

Similarly, the protein content of infant formula may also influence the infant gut microbiome [38]. High protein intake during early infancy has been suggested to alter the composition and function of the gut microbiota [39]. Protein is an essential macronutrient for growth and development; however, excessive protein intake, especially in the absence of adequate fiber and complex carbohydrates, may favor the growth of certain bacteria that thrive on protein breakdown [40]. This shift in the microbial composition could potentially lead to an imbalance in the gut microbiota and may have implications for immune development and long-term health outcomes [40].

## 5. Conclusions

In conclusion, our study highlights some potential nutritional consequences of selecting a lactose-reduced or lactose-free infant formula, when not medically indicated. Lactose-reduced and lactose-free formulas had higher protein, added sugars, GI, and certain PUFAs compared to lactose-based formulas. These modifications could have significant implications for infant growth, metabolic responses, and long-term health trajectories. As the nutritional needs of infants can vary based on individual factors, including age, weight, and health conditions, it is essential to carefully consider the nutrient content when selecting an appropriate infant formula to support optimal nutrition and development. The findings underscore the importance of understanding the nutritional changes that occur when lactose is replaced and the potential effects on infants’ various health outcomes such as obesity, metabolic function, immune function, and the establishment of a healthy gut microbiome. As lactose is the primary energy carbohydrate source in human milk, careful consideration of its substitutes is vital for providing optimal nutrition to infants. Further research is warranted to explore the long-term consequences of lactose replacement and to guide evidence-based recommendations for infant formula composition to support the health and well-being of infants. Our study primarily addresses the US market while acknowledging the potential for regional variations. This emphasizes the importance of considering specific regional contexts and warrants further investigation into international product distinctions. In addition, while this study provides valuable insights, it may not comprehensively encompass all the complexities of formula feeding. To address this gap, there is a compelling need for dedicated research that focuses specifically on formula feeding. Such studies will refine our understanding of how formula component changes affect formula-fed infants, enabling tailored dietary recommendations.

## Figures and Tables

**Table 1:** Comparison of nutrient composition per 100 g of formula between infant formulas (*n* = 88) with reference to human milk.

	100% Lactose *n* = 7	Reduced Lactose *n* = 37	Lactose Free *n* = 44	Human Milk *	*p*-Value
**Dietary Variables**					
glycemic index (glucose reference)	44.36 ± 1.65	62.5 ± 16.97	94.9 ± 7.48	41	<0.001
glycemic index (bread reference)	63.38 ± 2.34	89.28 ± 24.24	135.57 ± 10.68	58.63	<0.001
**Carbohydrate (g)**					
Sucrose	0 ± 0	0.25 ± 1.03	2.14 ± 2.41	0	<0.001
Added Sugars	2.41 ± 2.41	4.37 ± 1.63	5.29 ± 1.87	0	<0.01
Soluble Fiber	0 ± 0	0.14 ± 0.21	0.04 ± 0.11	0	0.01
Fiber	0 ± 0	0.14 ± 0.21	0.06 ± 0.13	0	0.02
**Protein (g)**					
Total Protein	1.38 ± 0.18	1.6 ± 0.33	1.84 ± 0.44	1.03	<0.001
Animal Protein	1.38 ± 0.18	1.51 ± 0.46	0.85 ± 0.99	1.03	<0.01
Vegetable Protein	0 ± 0	0.09 ± 0.28	0.99 ± 0.93	0	<0.001
**Fats (g)**					
Total PUFA	0.65 ± 0.08	0.68 ± 0.08	0.8 ± 0.22	0.49	<0.01
PUFA Linoleic Acid (LA)	0.5 ± 0.1	0.5 ± 0.1	0.7 ± 0.2	0.29	<0.01
Omega-6	0.5 ± 0.1	0.55 ± 0.1	0.67 ± 0.22	0.32	<0.01
Omega-3	0.06 ± 0.02	0.07 ± 0.01	0.08 ± 0.02	0.04	0.01
Linolenic Acid	0.05 ± 0.01	0.06 ± 0.01	0.07 ± 0.02	0.05	<0.01
Alpha Linolenic Acid (ALA)	0.05 ± 0.01	0.06 ± 0.01	0.07 ± 0.02	0.04	<0.01
Linoleic Acid	0.57 ± 0.1	0.59 ± 0.08	0.7 ± 0.21	0.37	<0.01
Stearic Acid	0.16 ± 0.06	0.13 ± 0.03	0.12 ± 0.04	0.29	0.02
Palmitic Acid	0.72 ± 0.24	0.52 ± 0.27	0.41 ± 0.24	0.91	<0.01
Palmitoleic Acid	0.02 ± 0.05	0.01 ± 0.01	0 ± 0.01	0.12	<0.01
**Micronutrients**					
Vitamin D	0.88 ± 0.36	1.35 ± 0.96	0.98 ± 0.12	0.07	0.03
Vitamin K	4.67 ± 1.96	5.56 ± 0.66	6.08 ± 1.39	0.3	<0.01
Riboflavin	0.09 ± 0.02	0.11 ± 0.04	0.08 ± 0.05	0.03	0.02
Vitamin B12	0.18 ± 0.06	0.2 ± 0.06	0.27 ± 0.13	0.05	<0.01
Sodium	19.17 ± 1.57	24.37 ± 7	29.78 ± 4.66	17	<0.001
Potassium	67.69 ± 11.63	74.34 ± 11.16	82.07 ± 21.79	51	0.03
Calcium	48.63 ± 9.56	75 ± 32.03	74.38 ± 17.22	32	0.03
Phosphorus	25.38 ± 6.64	42.48 ± 20.14	50.23 ± 16.51	14	<0.01
Magnesium	5.15 ± 1.1	5.89 ± 2.16	7.94 ± 4.61	3	0.01
Manganese	0.01 ± 0.01	0.01 ± 0.03	0.04 ± 0.08	0.02	0.03
Iron	0.95 ± 0.5	1.16 ± 0.2	1.22 ± 0.07	0.03	<0.01

Values are presented as Mean ± SD. *p*-values represent statistical significance for *p*-values < 0.05. The reported *p*-value reflects the outcome of an analysis conducted through linear regression, focusing on the association between the dietary variables and distinct formula-type categories. This analysis, which inherently involves ANOVA through linear regression, reveals potential variations in the nutrient composition of the formula groups. * The column representing nutrient values for human milk serves solely for visual comparison and is not subjected to statistical analysis, including the provided *p*-values. The values for human milk are referenced from NDSR.

**Table 2:** Regression analysis of different nutrients with lactose content.

Variable	β	(95% CI)	*p*-Value
glycemic index (bread reference)	−10.12	−10.87, −9.37	<0.001
glycemic index (glucose reference)	−7.09	−7.61, −6.55	<0.001
**Carbohydrate**			
Added Sugars	−0.19	−0.32, −0.05	<0.01
Sucrose	−0.33	−0.46, −0.20	<0.001
Fiber	0.01	0.004, 0.02	<0.01
Soluble Fiber	0.01	0.007, 0.03	<0.01
**Protein**			
Protein	−0.05	−0.08, −0.02	<0.001
Vegetable Protein	−0.15	−0.19, −0.10	<0.001
Animal Protein	0.09	0.04, 0.15	<0.001
**Fat**			
Total PUFA	−0.01	−0.02, −0.005	<0.01
Linoleic Acid	−0.01	−0.02, −0.005	<0.01
Arachidonic Acid	0.001	0.0002, 0.001	<0.01
Linolenic Acid	0.001	−0.003, 0.0006	<0.01
Alpha linolenic Acid	0.001	−0.003, −0.0005	<0.01
Omega-6	−0.01	−0.02, −0.004	<0.01
Omega-3	−0.001	−0.002, −0.0003	0.01
Palmitic Acid	0.01	0.0001, 0.03	0.04
**Vitamins and Minerals**			
Pantothenic Acid	−0.01	−0.02, −0.0003	0.04
B12	−0.01	−0.01, −0.005	<0.001
Vitamin K	−0.09	−0.18, −0.01	0.02
Magnesium	−0.41	−0.65, −0.17	<0.001
Sodium	−1.24	−1.65, −0.84	<0.001
Phosphorus	−2.46	−3.74, −1.18	<0.001
Manganese	−0.005	−0.009, −0.001	<0.01
Potassium	−1.66	−2.85, −0.48	<0.01
Iron	−0.01	−0.03, −0.0009	0.03

Beta coefficients and 95% confidence intervals (CIs) from linear regression analysis were used to examine the associations between gram lactose concentration with gram dietary nutrients and glycemic index. The table includes only statistically significant associations <0.05. The Beta coefficient represents the strength and direction of the linear relationship between lactose concentration and each nutrient or glycemic index value.

## Data Availability

An anonymized dataset including all data described in the manuscript, code book, and analytic code will be made available upon request to the principal investigator M.I.G.

## Supplementary Materials

The following supporting information can be downloaded at: https://www.mdpi.com/article/10.3390/nu16020276/s1, Figure S1: The Flow of Formula categorization and nutrient comparison.
