# Supplementary figures and images for: Altered Nutrient Composition of Lactose-Reduced Infant Formula
Source: Nutrients. 2024 Jan 17;16(2):276. doi: 10.3390/nu16020276 (PMC10821187; doi:10.3390/nu16020276)

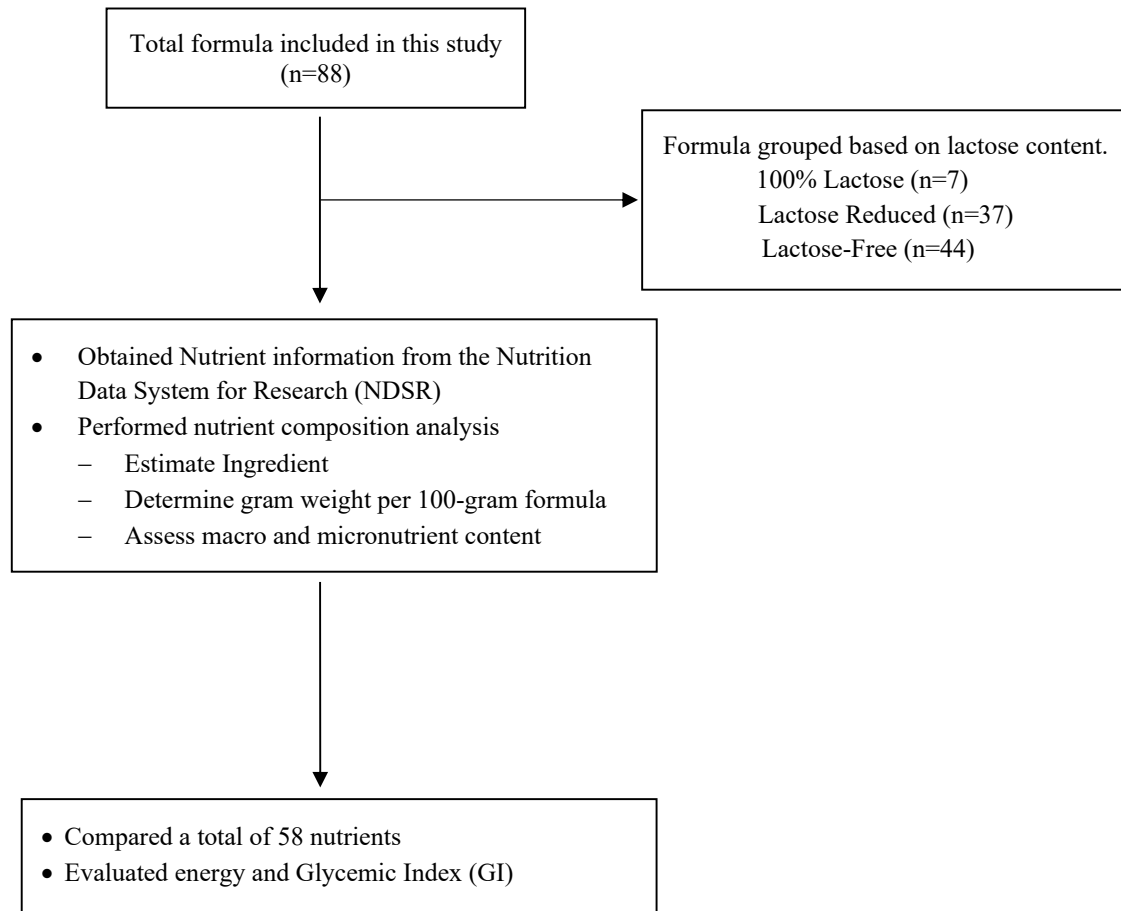

Figure S1. The Flow of Formula categorization and nutrient comparison

Supplement: Supplementary file 1 [file nutrients-16-00276-s001.zip › nutrients-2740453-supplementary.pdf]
